# Choice and consumption of animal or plant-based protein: Controlling experimentally and statistically for sensory stimulus aspects and food choice factors

**DOI:** 10.1016/j.mex.2021.101557

**Published:** 2021-10-27

**Authors:** Attila Pohlmann

**Affiliations:** Universidad San Francisco de Quito USFQ, Ecuador

**Keywords:** Protein choice, Consumer behavior, Food choice behavior, Nutritional information, Physical characteristics, Sensory food properties, Food health perceptions, Meat consumption, Vegetarianism

## Abstract

Everyday consumer food choices are influenced by a variety of interacting biological, situational, economical, and psychological factors [1], [2], [3], [4]. The choice between animal-based and plant-based protein has implications for overall and cause-specific mortality and cardiometabolic health (e.g. [5], [6], [7], [8], [9]). During laboratory experiments that are designed to better understand factors that influence protein choice specifically, controlling for the sensory aspects of stimuli, health information, consumers’ physical characteristics, and dietary preferences is crucial. To illustrate the point, if a choice task included two stimuli, brown rice with tofu and steak with fries for example, a variety of factors, such as visual appeal and hedonic attributes could influence protein choice and dilute the effect of the experimental manipulation. This article provides a template for a generic experiment to measure participant choice among salt-cured jerky protein sources (e.g. beef, salmon, soy, textured vegetable protein, turkey, tuna) and consumed amounts. Using jerky products as stimuli minimizes variance in visual appearance, texture, and nutritional values, therefore facilitating the attribution of the experimental factor(s). A list of methods to experimentally and/or statistically control for potential sources of measurement error is provided.

• Consumer choice of animal vs. plant-based protein has implications for individual and environmental health.
• The methods can be used to customize experiments in consumer behavior research, psychology, and nutrition sciences.
• Food choice is influenced by a variety of factors; experimentally and/or statistically controlling for major sources of measurement error increases confidence in the effect of the manipulated variable.

## Graphical abstract

**Figure:**
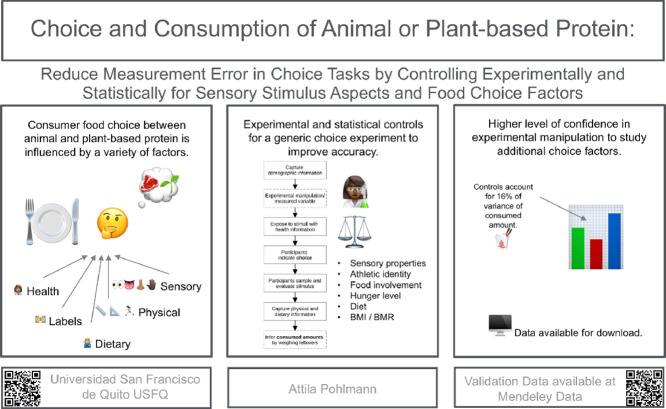

Specifications table

**Table:** 

Subject Area:	Psychology
More specific subject area:	Psychology of Meat Consumption
Method name:	Controlling experimentally and statistically for sensory stimulus aspects of animal and plant-based protein sources.
Name and reference of original method:	Eating habits, food selection [1], [2], [3], [4]
Resource availability:	Validation Data available on Mendeley Data [10]

## Method details

**Fig. 1:**
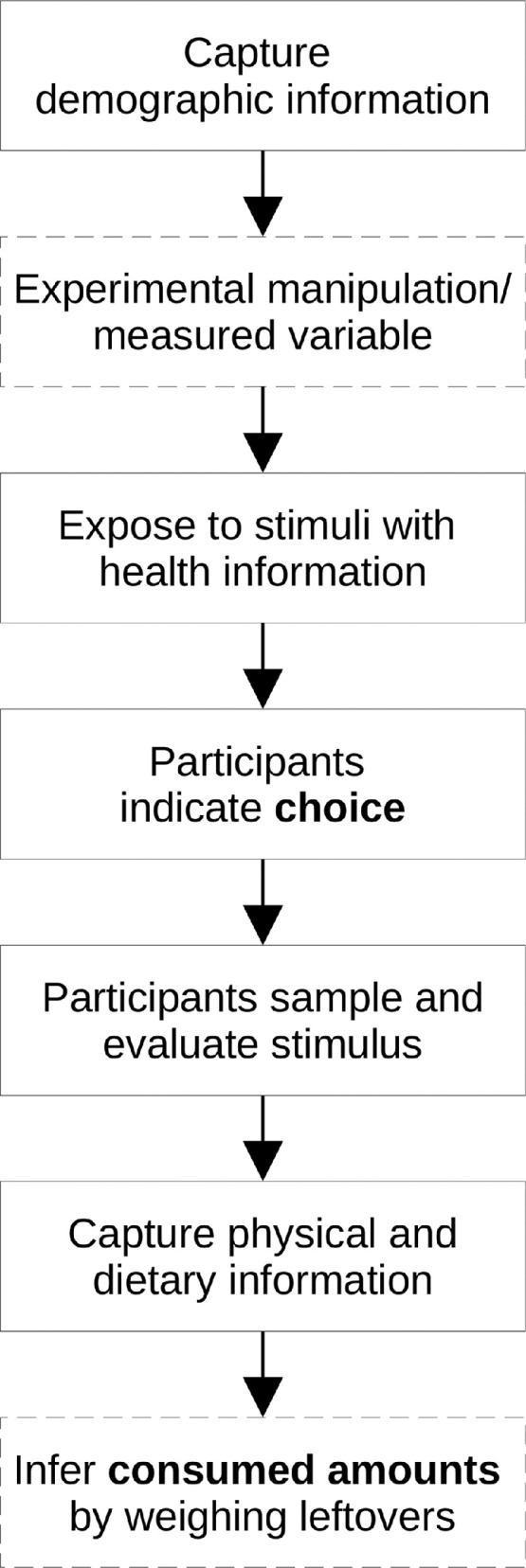
Suggested steps in a generic choice experiment.

**Fig. 2:**
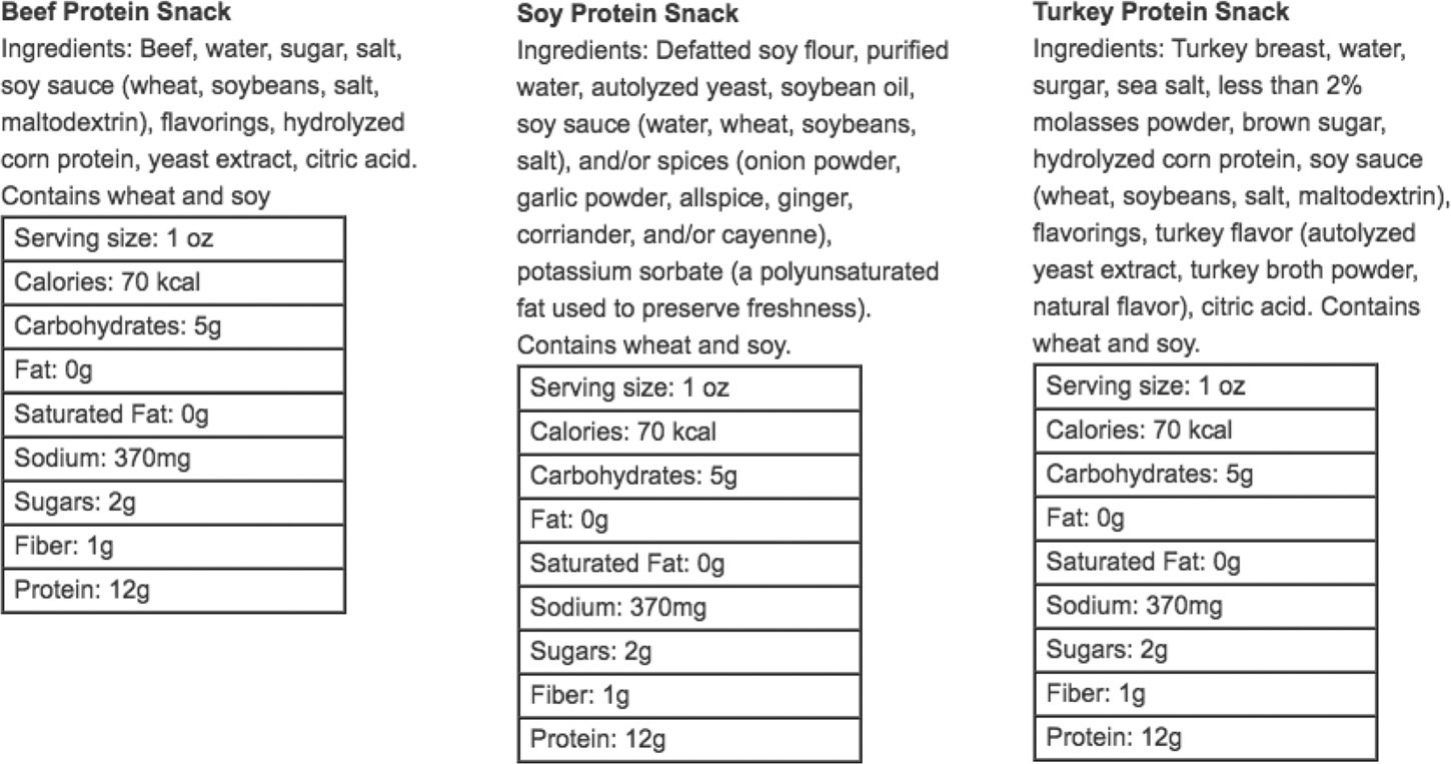
Example of health information to be provided with stimuli.

Fig. 1 illustrates the sequence of steps of a generic choice experiment, Table 1 provides a list of of stimulus aspects that can be experimentally and/or statistically controlled for. Fig. 2 illustrates an example of health information that is made to be near-identical for each stimulus in the choice task.

**Table 1:** List of experimental and statistical controls.

Sensory		
Appearance	Choose visually similar experimental stimuli that only vary on main protein source. Jerky products are available in various versions: meat (e.g. beef, turkey), seafood (e.g. tuna, salmon), and vegetarian/vegan (e.g. soy jerky, textured vegetable proteins).	Adjective pairs for semantic differentials (Osgood, 1964): 3 = “looks bad,” 3 = “looks good,” looks repulsive/looks appealing, looks inedible/looks edible.
Texture	Jerky products are comparable in texture. Tactile exposure can be delayed until after participants indicate their choice.	3 = “hard,” 3 = “tender,” chunky/minced, dry/moist.
Aroma	Olfactory exposure can be delayed after choice is made. Provide stimulus in sealed container/bags.	3 = “smells bad,” 3 = “smells good.”
Taste	Pretest jerky products for differences in taste.	3 = “tastes bad,” 3 = “tastes good,” flavorless/flavorful, unpalatable/scrumptious, disgusting/delicious, yuck/yum.
Health		
Ingredients	Choose jerky products that have similar ingredient lists and only vary on main protein.	Processing covariate can be added, e.g. “I am familiar with this type of nutritional labels.” “I am proficient with reading nutritional labels.” (1 = “strongly disagree,” 5 = “strongly agree”)
Calories	Choose equal portion sizes and average calorie amounts across stimuli. Label each item with same caloric value, see Fig. 2.	
Nutritional information	Average nutritional components such as carbohydrates, fat, sugar, protein across stimuli. Label each item with same values, see Fig. 2.	
Labels		
Convenience	Limited relevance in laboratory studies.	-
Process (time, effort)	Limited relevance in laboratory studies.	-
Cost	Unless focal variable, of limited relevance in laboratory studies.	-
Physical		
Biological sex and age	Capture at beginning of survey.	-
Height and weight	Capture at end of survey to prevent potential body image priming, which could otherwise influence subsequent food choice and consumption amounts.	Composite variables such as Body Mass Index (BMI) and predicted Base Metabolic Rate (BMR) can be calculated to statistically adjust for base consumption levels.
Physical activity	Prescreen participants or use as exclusion criterion.	Various scales are available to measure levels of physical activity (Cieslak, 2004; Sylvia et al., 2014)⁠. Higher levels of physical activity generally require larger amounts of energy and influence protein choice and intake (Phillips, 2012).⁠
Dietary		
Hunger level	Aim to survey participants during same time of day.	Approximate hunger level by asking how many hours ago a participant had last eaten a snack or a meal (Moskowitz et al., 1976)⁠.
Calorie restrictive diet	Prescreen participants or use self-reports as exclusion criterion.	Survey participants whether they are on a calorie-restrictive diet. E.g. Atkins, Weight Watchers, calorie-counting, fasting, etc.
Food involvement	-	Individual psychological differences in food involvement (Bell and Marshall, 2003)⁠, which influences information search, information processing, and choice.
Meat avoidance intent	-	Measure meat avoidance intent (Rozin et al., 2012)⁠ without using meaning-laden and potentially ambiguous labels such as vegetarian, vegan, or flexitarian.

The first step in the experiment is to capture demographic information and perhaps other enduring psychological traits that are pertinent to the hypotheses. Subsequently, the experimental manipulation is administered or the independent variable is measured. Jerky products with similar ingredients, which vary primarily on the main protein sources, should be chosen as experimental stimuli, if consumer protein choice is the variable of interest. Jerkies are visually similar and are commercially available in various meat, seafood, vegetarian, and vegan versions. If consumption amounts will be measured, the stimuli should be pretested to ensure that no significant differences in taste exist among the products.

Fig. 2 shows an example of health information for three jerky products (beef, turkey, soy) that participants should be exposed to before the choice task. If the function is provided by the survey software, the display order in which this information is provided should be randomized. This step ensures that health and nutritional information is not only absorbed by participants, but also presented as ostensibly near-identical, therefore standardizing health-relevant information. In essence, since nutritional information is deliberately presented as near-identical, the influence of health concerns on choice is minimized. Aside from health information, convenience, process, and cost are factors in consumer food choices, but they are less relevant in laboratory studies [4]. Portion sizes should be equal; the presented caloric amounts and nutritional components (carbohydrates, fat, protein, etc.) should be averaged from the actual values that are provided with the product by the manufacturer. During this step, processing covariates can be employed, such as “I am familiar with this type of nutritional labels.” “I am proficient with reading nutritional labels.” (1 = “strongly disagree,” 5 = “strongly agree”), or simply adding a background timer that measures how long participants are exposed to the nutritional information, which can be used as proxy for engagement with and absorption of the information. The principal investigator should clear with the IRB whether the averaging of nutritional information is considered deceptive and, if need be, make adjustments accordingly. Also, participants should be screened for allergies if stimuli will be consumed and the full list of ingredients provided by the manufacturer should be disclosed.

After this step, participants make a choice and receive a serving of their chosen item for sampling. Up to this point, visual and olfactory perceptions should be limited since they could lead participants to make inferences about sensory aspects prematurely and bias their choice. Once participants receive the actual serving of the chosen stimulus–ideally in a sealed container–they evaluate its sensory properties: Table 2 provides a list of positively and negatively valenced, food-related adjective pairs that pertain to the sensory aspects of appearance, texture, aroma, and taste; e.g. (-3 = “tasted bad,” 3 = “tastes good”) These pairs can be used in semantic differentials [11] to statistically control for specific sensory aspects. Alternatively, the same semantic differentials can be employed as composite outcome variables.

**Table 2:** Descriptive statistics and PCA results for sensory properties items.

M	(SD)	Item	Factor 1	Factor 2
1.64	(1.24)	yuck/yum	**.90**	
1.50	(1.20)	disgusting/delicious	**.89**	
1.64	(1.22)	looks bad/good	**.88**	
1.79	(1.09)	tastes bad/good	**.82**	
2.17	(1.13)	looks inedible/edible	**.79**	
1.17	(1.10)	unpalatable/scrumptious	**.78**	
1.75	(1.12)	flavorful/flavorless	**.76**	
1.39	(1.18)	looks repulsive/appealing	**.72**	
1.44	(1.19)	smells bad/good	**.71**	
.35	(1.60)	hard/tender		**.85**
-.46	(1.77)	chunky/minced		**.76**
.02	(1.79)	dry/moist		**.76**
		*Cronbach's Alpha (factor)*	*.93*	*.70*
		*Cronbach's Alpha (total)*	*.86*	

Note: factor loadings smaller than .40 not shown

After participants are dismissed, consumed amounts can be inferred by weighing leftovers and associating them with survey responses (e.g. using a code printed on the underside of a plate or inconspicuously attached to the container). Since physical characteristics of consumers influence food choice and consumed amounts, these characteristics should be measured after the choice and evaluation/sampling tasks to avoid body image related priming effects. Composite covariates such as Body Mass Index (BMI) and predicted Basal Metabolic Rate (BMR) can be calculated by using self-reported information on biological sex, age, height, and weight. Additionally, information on physical activity should be captured since people who are physically active, generally have higher caloric requirements and/or different preferences for sources of protein [12]. See [13] for a list of suitable self-report instruments to measure physical activity or use the athletic identity scale provided in [14].

Finally, dietary factors influence participant food choice as well as consumption and should be controlled for. Needless to say, hunger level impacts appetite and subjective taste [15]: a covariate that approximately captures hunger level can be created by asking participants, at the beginning of the survey, how many hours ago they ate a meal or snack (0 = “less than an hour ago,” 1 = “one hour ago,” etc.; up to 5 h). Surveying participants at the same time of day also helps to ensure relatively similar hunger levels. Participants should indicate whether they are on any type of restrictive diet and ideally provide additional information in a comment field. Individual psychological differences in food involvement [16] indicate how consumers go about product information processing and choice. Dietary preferences based on moral and/or health reasons should be accounted for by capturing meat avoidance intent (MAI). Meaning-laden and potentially ambiguous labels such as omnivore, vegetarian, vegan, or flexitarian should be avoided. Instead, participants respond to three statements: 1 = yes, 0 = no; “I avoid eating red meat,” “I avoid eating meat: any animal flesh, e.g. beef, pork, seafood, chicken, etc.,” and “I avoid eating any product that comes from an animal,” adapted from [17]. The MAI variable is generated by summing the affirmative statements; a score of zero indicates no meat avoidance intent (e.g. omnivore), whereas a score of three indicates the highest possible level of intent to avoid animal products (e.g. vegan). The logical consistency of the statements should be checked manually or programmed into the survey software, i.e. preventing contradictory answers. This set of questions can be followed up with a self-report item on dietary pattern adherence [18].

## Method validation

One hundred and one participants (43 female) with a mean age of 22 years were recruited to sample a beef jerky in a classroom setting. Using the Qualtrics platform, they entered demographic information and indicated their hunger level. After sampling the jerky, participants indicated their height and weight, filled out the athletic identity scale [14], the food involvement scale [16], and indicated whether they were on a diet (0 = no, 1 = yes). Each participant received a sealed bag that contained one ounce (28 g). After participants were dismissed, the consumed amounts were inferred (*M* = 21.67, SD = 7.99) by weighing the leftovers. The consumed amounts were associated with survey responses using a unique, printed, nine-digit code that participants had received with their sample.

First, participants rated the jerky's sensory properties using 12 semantic differentials anchored with negatively and positively valenced adjectives: -3 = “tasted bad,” 3 = “tastes good,” yuck/yum, disgusting/delicious, looks bad/good, tastes bad/good, looks inedible/edible, unpalatable/scrumptious, flavorless/flavorful, looks repulsive/appealing, smells bad/ good, hard/tender, chunky/minced, dry/moist. The resulting scores were entered into a principal component analysis (PCA) with varimax rotation. To evaluate factor loadings, commonly accepted criteria were used: retain factors with a minimum eigenvalue of one [19], at least three items per factor [20], and a primary factor loading of at least .60 without cross-loading more than .40 on any other factor [21]. After the PCA, all factors were retained, which cumulatively account for 65.84% of variance. Descriptive statistics and factor loadings are shown in Table 2. The first factor combines the sensory aspects of appearance, aroma, and taste (9 items, Cronbach's alpha = .93). The second factor refers to texture (3 items, Cronbach's alpha = .70). The reliability of the total item scale is very good (12 items, Cronbach's alpha = .86).

In order to evaluate the effect of the sensory properties of the stimulus–using the total item scale–(*M* = .79, SD = .89), participant hunger level (*M* = 2.58, SD = 1.62), food involvement (*M* = 3.18, SD = .38), athletic identity (*M* = 3.15, SD = 1.06), diet (*M* = .16, SD = .37), and composite variables, such as BMI (*M* = 24.63, SD = 5.47), and predicted BMR (*M* = 1662.24, SD = 355.85), the amount of snack consumed was regressed on these criteria. Jointly, they account for 16% of the variance in the model. The combined sensory properties variable is a significant predictor of consumption amount (β = .22, SE = .89, *t* = 2.19, *p* = .031). Athletic identity is a marginally significant predictor (β = .21, SE = .79, *t* = 1.98, *p* = .051). The other predictors in the model are not significant at the .05 level, but this fact does not diminish their suitability as potential covariates (Hunger level: β = .05, SE = .49, *t* = .47, *p* = .640, N.S.; Food involvement: β = -.02, SE = 2.05, *t* = .16, *p* = .870, N.S.; Diet: β = .16, SE = 2.17, *t* = 1.57, *p* = .120, N.S.; BMR: β = .22, SE = .01, *t* = 1.22, *p* = .226, N.S.; BMI: β = -.14, SE = .24, *t* = -.84, *p* = .401, N.S.). The data used for validation and scripts for analysis are available on Mendeley Data [10]

The list of experimental and statistical methods to control for a variety factors that affect protein choice and consumption provided here is not exhaustive. Cultural factors greatly influence food choice and need to be taken into account depending on the specific research context. For instance, Indian vegetarians consider meat as polluting and associate abstaining from it with purity, authority, and in-group cohesion. These differences were not observed in Euro-Canadian and Euro-American vegetarians who are more concerned with animal welfare and the environmental impact of their diet [22]. As in the planning of every experiment, the principal investigator has to find a balance among practical considerations in terms of time allotted for the survey, availability of laboratory space or comparable alternative settings, and being able to test the main hypotheses. Naturally, the point of providing methods for experimental and statistical controls is not to explain away all the variance in food choice and consumption, but to reduce overall measurement error and to account for variance that can be attributed to known and tested theoretical factors. In the end, it may be impractical to include all covariates in the model to test the experimental hypotheses. Covariates should be evaluated for correlation and raw data should always be carefully checked before hypothesis tests are performed.

## Direct submission or co-submission

Co-Submission: Pohlmann, A. (2021). Lowering barriers to plant-based diets: The effect of human and non-human animal self-similarity on meat avoidance intent and sensory food satisfaction. Food Quality and Preference, 93, 104272.

## Declaration of Competing Interest

The author declares that he has no known competing financial interests or personal relationships that could have appeared to influence the work reported in this paper.

## Appendix Supplementary materials

Image, application 1

Image, application 2
